# Radiological Findings in SARS-CoV-2 Viral Pneumonia Compared to Other Viral Pneumonias: A Single-Centre Study

**DOI:** 10.1155/2022/2826524

**Published:** 2022-09-29

**Authors:** Rana Günöz Cömert, Eda Cingöz, Sevim Meşe, Görkem Durak, Atadan Tunaci, Ali Ağaçfidan, Mustafa Önel, Şükrü Mehmet Ertürk

**Affiliations:** ^1^Istanbul University, Istanbul Faculty of Medicine, Department of Radiology, Istanbul, Turkey; ^2^Istanbul University, Istanbul Faculty of Medicine, Department of Medical Microbiology, Istanbul, Turkey

## Abstract

**Background:**

Thorax computed tomography (CT) imaging is widely used as a diagnostic method in the diagnosis of coronavirus disease 2019 (COVID-19)-related pneumonia. Radiological differential diagnosis and isolation of other viral agents causing pneumonia in patients have gained importance, particularly during the pandemic.

**Aims:**

We aimed to investigate whether there is a difference between CT images from patients with COVID-19-associated pneumonia compared to CT images of patients with pneumonia due to other viral agents and which finding may be more effective in diagnosis. *Study Design*. The study included 249 adult patients with pneumonia identified by thorax CT examination and with a positive COVID-19 RT-PCR test compared to 94 patients diagnosed with non-COVID-19 pneumonia (viral PCR positive but no bacterial or fungal agents detected in other cultures) between 2015 and 2019. CT images were retrospectively analyzed using the PACS system. CT findings were evaluated by two radiologists with 5 and 20 years of experience, in a blinded fashion, and the outcome was decided by consensus.

**Methods:**

Demographic data (age, gender, and known chronic disease) and CT imaging findings (percentage of involvement, number of lesions, distribution preference, dominant pattern, ground-glass opacity distribution pattern, nodule, tree in bud sign, interstitial changes, crazy paving sign, reversed halo sign, vacuolar sign, halo sign, vascular enlargement, linear opacities, traction bronchiectasis, peribronchial wall thickness, air trapping, pleural retraction, pleural effusion, pericardial effusion, cavitation, mediastinal/hilar lymphadenopathy, dominant lesion size, consolidation, subpleural curvilinear opacities, air bronchogram, and pleural thickening) of the patients were evaluated. CT findings were also evaluated with the RSNA consensus guideline and the CORADS scoring system. Data were divided into two main groups—non-COVID-19 and COVID-19 pneumonia—and compared statistically with chi-squared tests and multiple regression analysis of independent variables.

**Results:**

RSNA and CORADS classifications of CT scan images were able to successfully differentiate between positive and negative COVID-19 pneumonia patients. Statistically significant differences were found between the two patient groups in various categories including the percentage of involvement, number of lesions, distribution preference, dominant pattern, nodule, tree in bud, interstitial changes, crazy paving, reverse halo vascular enlargement, peribronchial wall thickness, air trapping, pleural retraction, pleural/pericardial effusion, cavitation, and mediastinal/hilar lymphadenopathy (*p* < 0.01). Multiple linear regression analysis of independent variables found a significant effect in reverse halo sign (*β* = 0.097, *p* < 0.05) and pleural effusion (*β* = 10.631, *p* < 0.05) on COVID-19 pneumonia patients.

**Conclusion:**

The presence of reverse halo and absence of pleural effusion was found to be characteristic of COVID-19 pneumonia and therefore a reliable diagnostic tool to differentiate it from non-COVID-19 pneumonia.

## 1. Introduction

Viruses are the most common cause of respiratory tract infections. It has been reported that viruses such as influenza, human parainfluenza viruses (HPIVs), adenovirus, respiratory syncytial virus (RSV), and human metapneumovirus (HMPV) can cause lower respiratory tract infections in individuals with both normal immune systems and immunodeficiency. Studies show that viruses such as rhinovirus, endemic coronaviruses, cytomegalovirus (CMV), herpes simplex virus (HSV), varicella zoster virus (VZV), and human bocavirus (HBoV) can cause lower respiratory tract infection only in those with immunodeficiency [1].

It is reported that COVID-19 infection can be examined in 3 stages as follows: the first is the asymptomatic period, the second is the upper and lower respiratory tract response, and the third is the widespread lung involvement that can progress to acute respiratory distress syndrome [2]. Approximately 80% of the patients with COVID-19 are asymptomatic or limited to mild to moderate symptoms in the first two stages. In the remaining 15 to 20% of the patients, pulmonary ground glass opacity consolidation is detected as a radiological finding due to the inflammatory response in the lung [2].

If there is no risk factor for the progression of the COVID-19 disease in patients with mild clinical symptoms, there is no imaging indication, and imaging should be performed in cases with worsening respiratory system symptoms. Imaging can be performed to provide medical triage in cases with the patient is suspected to have COVID-19 with moderate-to-severe symptoms if clinical conditions require it [3].

A normal chest X-ray does not exclude COVID-19 pneumonia, especially in cases with mild pneumonia or during early-stage disease [3, 4]. CT scans cannot be used as a screening test since the positive predictive value of thoracic CT in the diagnosis of COVID-19 is 92% while the negative predictive value is 42% [5], and the absence of CT findings in the early phase of disease should not exclude the possibility of COVID-19 disease [6, 7]. Furthermore, the combination of repeated RT-PCR tests and thoracic CT examination is beneficial in cases with suspected COVID-19 [8].

CT imaging results of viral pneumonia may overlap with nonviral infections and inflammatory conditions. Some diagnostic patterns of viral pneumonia help to make differential diagnoses in the early stages of infection, reduce unnecessary antibiotic use, and prevent disease spread [1]. In a thorax CT of a patient with viral pneumonia, reticular opacities caused by interstitial inflammation, ground-glass opacity (GGO) due to alveolar edema, patchy consolidation, localized atelectasis, peribronchovascular thickening, centrilobular nodular opacities, tree in bud pattern, and interlobular septal thickening are visible, but it is reported that diagnosis cannot be made based on imaging findings alone [9, 10]. However, the detection of centrilobular nodular opacities, pleural effusion, and lymphadenopathy more frequently in non-COVID-19 viral pneumonia has been reported to help differential diagnosis [10].

Thorax CT imaging is commonly used for the diagnosis of COVID-19. During the COVID-19 pandemic, radiological differential diagnosis of seasonal epidemics in immunocompromised patients or other viral agents causing pneumonia in immunosuppressed patients has become increasingly important for early diagnosis and isolation. Therefore, we aimed to investigate the differences between CT image findings characteristic of COVID-19 pneumonia patients and CT findings from patients with pneumonia caused by other viral agents.

## 2. Materials and Methods

### 2.1. Researched Patient Population

249 COVID-19 patients aged 18 years and older, who were admitted to our hospital between March 15, 2020, and May 30, 2020, tested positive for the virus by RT-PCR of nasopharyngeal swab samples taken at the application stage, and pneumonia was detected from thorax CT examination at admission. Other viral agents that could cause pneumonia were excluded from the respiratory viral panel of the COVID-19 patient group.

The non-COVID-19 group consists of patients aged 18 and above with the viral respiratory panel or bronchoalveolar lavage/blood viral PCR results within an average of 5.67 ± 7.95 days, between January 2015 and December 2019 (data from the last 5 years before the onset of the COVID-19 pandemic was scanned). The thorax CT findings from these patients were compatible with viral pneumonia as 94 patients were positive, but no bacterial or fungal agents were detected in other sputum and blood cultures (viral panel results: influenza, A-B *n* = 26; adenovirus, *n* = 5; CMV, *n* = 28; RSV, *n* = 8; parainfluenza, *n* = 10; and HMPV, *n* = 3); endemic coronaviruses (HCoV-NL63, HCoV-HKU, HCoV-229E, and HCoV-OC43, *n* = 16; rhinovirus, *n* = 7; and human bocavirus (HBoV) *n* = 1) were included in the study. In the non-COVID-19 group, there were cases in which more than one agent was found together in the respiratory viral panel.

### 2.2. Laboratory PCR Test Method

The FTD Respiratory Pathogens 21 kit (fast-tract DIAGNOSTICS, Luxembourg), which is based on the reverse transcriptase multiplex PCR method, was used for the viral respiratory panel. Artus CMV QS-RGQ kit QIAsymphony RGQ system (QIAGEN, Germany) was used as a CMV DNA quantitative test for patients between January 2015 and September 2018 (measuring range of the kit: 79.4 copies/mL-100,000,000 copies/mL, 1 copy/mL = 1.64 IU/mL). COBAS AmpliPrep/TaqMan CMV test, and COBAS AmpliPrep/TaqMan system were used for patients between September 2018 and December 2019 (measuring range of the kit: 150 copies/mL-10000000 copies/mL, 1 copy/mL = 0.91 IU/mL).

Viral RNA extraction from respiratory samples of patients with COVID-19 symptoms was performed manually with Bio-Speedy® Viral Nucleic Acid Isolation Kit (Bioeksen R&D Technologies Company, Turkey). RT-qPCR was performed on the Rotor-Gene *Q* 5 Plex Real-Time PCR (Qiagen, Germany) using Bio-Speedy® COVID-19 RT-qPCR Detection Kit (Bioeksen Ar-Ge Technologies Company, Turkey). In the working principle of this kit, the human ribonuclease P (RNase P) gene is targeted as an internal control. The positivity of RNAse P allows for the evaluation of the RT-qPCR process by confirming the extraction process, and the SARS-CoV-2 PCR result is interpreted as positive with the detection of the amplification curve of the RdRp gene region.

### 2.3. Thorax CT Examination Protocol, Evaluation, and Statistical Analysis

Thorax CT examination protocol: tube voltage, 120 kV with 64 detectors (Aquillion, Toshiba) and 16 detectors (Brilliance, Philips); tube current modulation, 50 to 150 mA; range, 0.85 to 1.4; image slice thickness, 1 to 5 mm, CT images obtained in the supine position in full inspiratory in all patients and −600 to +1600 HU for lung parenchyma, +50 to +350 HU for mediastinum using window width retrospectively analyzed using the PACS System. CT findings were evaluated by two radiologists with 5 and 20 years of experience, in a blinded fashion, and the final decision was made by consensus.

The age, gender, and known chronic diseases of the patients were taken into consideration. CT image considerations including the percentage of involvement, number of lesions, distribution preference, dominant pattern, GGO distribution pattern, nodule, tree in bud sign, interstitial changes, crazy paving sign, reversed halo sign, vacuolar sign, halo sign, vascular enlargement (vascular structures with increased calibration relative to the proximal, which is thought to be due to mediators that cause hyperemia, in the area of inflammation or in the periphery of the lesion [11]), linear opacities, traction bronchiectasis, peribronchial wall thickness, air trapping, pleural retraction, pleural effusion, pericardial effusion, cavitation, mediastinal/hilar lymphadenopathy, dominant lesion size, consolidation, subpleural curvilinear opacities, air bronchogram, and pleural thickening were examined. CT findings were also evaluated with the RSNA consensus guidelines and the CORADS scoring system; data obtained were divided into two main groups—non-COVID-19 pneumonia and COVID-19 pneumonia—and statistically compared using chi-square tests and multiple regression analysis of independent variables.

## 3. Results

In the study, age ranged between 18 and 91, with a mean of 51.99 ± 16.99, with a median value of 53. The age of the non-COVID-19 patient group ranged from 18 to 84, with a mean of 49.29 ± 19.43. The age of the COVID-19 patient group ranged from 18 to 91, with a mean of 53.01 ± 15.91. In the study, 59.5% (*n* = 204) of the patients were male and 40.5% (*n* = 139) were female. 58.5% (*n* = 55) of the non-COVID-19 pneumonia patient group were male; 41.5% (*n* = 39) were female. 59.8% (*n* = 149) of the COVID-19 pneumonia patient group were male; 40.2% (*n* = 100) were female (Table 1).

33% (*n* = 113) of the COVID-19 patient group had no chronic disease. Compared to the COVID-19 group, the non-COVID-19 group (*n* = 94) all had chronic diseases. Concomitant chronic diseases of COVID-19 patients include cardiovascular disease (4.1%, *n* = 14, vs. 3.7%, *n* = 4), hypertension (22.5%, *n* = 77, vs. 1.9%, *n* = 2), diabetes mellitus (14.6%, *n* = 50, vs. 5.6%, *n* = 6), chronic lung disease (1.8%, *n* = 6, vs. 2.8%, *n* = 3), chronic liver disease (0%, *n* = 0, vs. 1.9%, *n* = 2), chronic kidney disease (2.3%, *n* = 8, vs. 19.4%, *n* = 21), extrapulmonary malignancy (3.2%, *n* = 11, vs. 21.3%, *n* = 23), conditions related to immunodeficiency (3.5%, *n* = 12, vs. 28.7%, *n* = 31), and others (14.9%, *n* = 51, vs. 14.8%, *n* = 16) compared to non-COVID-19 patients (Table 1).

Compared to the non-COVID-19 group, the COVID-19 group showed significantly higher percentages for RSNA typical group and CORADS 5 score (*p* < 0.01). Non-COVID-19 patients showed higher percentages for RSNA indeterminate group, CORADS 3 score, and CORADS 2 score compared to COVID-19 patients (*p* < 0.01), while there was no significant difference with the CORADS 4 score (Table 2).

Compared to the non-COVID-19 group, the COVID-19 group showed significantly higher percentages for peripheral distribution (40.7%, *n* = 101, vs. 11.7%, *n* = 11), dominant pattern of lung involvement is ground glass opacity (78.7%, *n* = 196, vs. 56.4%, *n* = 53), peripheral-bilateral distribution pattern of GGO (56.2%, *n* = 140, vs. 20.2%, *n* = 19), fine reticular opacity (40.2%, *n* = 100, vs. 31.9%, *n* = 30), crazy paving pattern (30.5%, *n* = 76, vs. 13.8%, *n* = 13), reversed halo (43.8%, *n* = 109, vs. 6.4%, *n* = 6), and microvascular enlargement (83.1%, *n* = 207, vs. 63.8%, *n* = 60) (*p* < 0.01) (Table 2).

Compared to the COVID-19 group, the non-COVID-19 group showed significantly higher percentages for peribronchial wall thickening (32.9%, *n* = 82, vs. 58.5%, *n* = 55), air trapping (11.6%, *n* = 29, vs. 33%, *n* = 31), pleural retraction (39.8%, *n* = 99, vs. 57.4%, *n* = 54), pleural effusion (3.2%, *n* = 8, vs. 33%, *n* = 31), pericardial effusion (3.6%, *n* = 9, vs. 29.8%, *n* = 28), cavitation (0%, *n* = 0, vs. 3.2%, *n* = 3), mediastinal lymph node nonspecific (92%, *n* = 229, vs. 69.1%, *n* = 65), pathological (8%, *n* = 20, vs. 29.8%, *n* = 28), and another reason (0%, *n* = 0, vs. 1%, *n* = 1) (*p* < 0.01) (Table 2).

Multiple linear regression analysis was performed to determine the effect of independent variables on COVID-19 pneumonia, and it was found that those with reversed halo sign (*β* = 0.097, *p* < 0.05) and those with pleural effusion (*β* = 10.631, *p* < 0.05) had a significant effect on COVID-19 pneumonia, whereby the presence of reversed halo sign and absence of pleural effusion was found to be efficient in the diagnosis of COVID-19 pneumonia patients (Table 3).

## 4. Discussion and Conclusion

In viral pneumonia, the pathogenesis of the agent, the age and immunity of the patient, and the presence of bacterial coinfection all affect thorax CT findings. Viruses with cytopathic effects such as influenza, CMV, and adenovirus can cause a characteristic lung injury pattern distinguishable by CT scan. Influenza invades the respiratory epithelium and causes perialveolar inflammation, including bronchi, exudate, increased peribronchial wall thickness, necrotizing bronchopneumonia, diffuse alveolar damage, clustered GGO around the bronchial tree, and large areas of consolidation that rapidly coalesce even in the early stages, which are formed and visible by CT scan [1]. After replicating in the nasopharyngeal epithelium, adenovirus, RSV, and HPIV begin their distribution to the lung by affecting the small airways and causing bronchiolitis. These viruses cause a characteristic bronchocentric appearance, but they can form multilobar diffuse consolidation in immunosuppressed patients [1]. CMV affects the airway and alveolar epithelium, causing GGO, interstitial pneumonia, and miliary nodules in areas of diffuse alveolar damage [12]. HMPV initiates inflammation by directly infecting the alveolar epithelium and produces GGO in faintly circumscribed nodules and immunosuppressed patients [1]. SARS-CoV-2 causes damage, exudate accumulation, and proliferation, especially in more peripheral alveoli, resulting in multifocal and peripherally distributed GGO, and consolidation findings in prolonged disease course depending on the severity of the alveolitis [13, 14].

### 4.1. The Gold Standard Method for Screening and Diagnosis of COVID-19 Is the RT-PCR Test

Since the thorax CT examination is the most commonly used method in clinical practice after RT-PCR, it was aimed to investigate whether the characteristic imaging findings diagnosed for COVID-19 pneumonia and classification systems established for the standardization of these findings differ from the CT findings detected in pneumonia caused by other viral agents.

**Figure 1:**
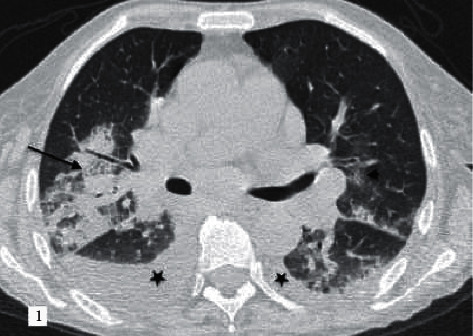
A 70-year-old female patient diagnosed with HCoV-OC43 pneumonia and chronic lymphocytic leukemia (CLL). According to the RSNA guidelines, CORADS score is given as 5. GGO (crazy paving) (black arrow) accompanied by interlobular and intralobular septal thickening on the axial CT section and patchy consolidation areas, faint GGO areas (black arrowhead), and pleural effusion (asterisks).

Pleural effusion is a more common finding in non-COVID-19 viral pneumonia than in COVID-19 pneumonia [10] (Figure 1). Although this information supports our results, in our study, all of the patients with non-COVID-19 viral pneumonia had concomitant chronic diseases, while 33% (*n* = 113) of the patient group with COVID-19 pneumonia had no chronic disease. The higher prevalence of diseases such as cardiovascular disease, chronic renal failure, extrapulmonary malignancy, and immunodeficiency-related conditions (73.1% vs. 13.1%) in the non-COVID-19 viral pneumonia patient group also contributed to significant pleural effusion as demonstrated by regression analysis (Table 3).

**Figure 2:**
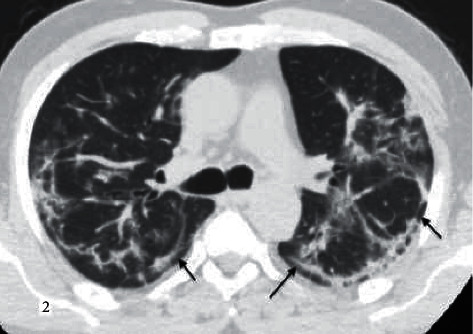
A 55-year-old male patient with COVID-19 pneumonia and known history of hypertension. “Typical” according to RSNA guidelines, and CORADS score given as 5. Bilateral widespread subpleural curvilinear opacities are demonstrated (black arrows).

**Figure 3:**
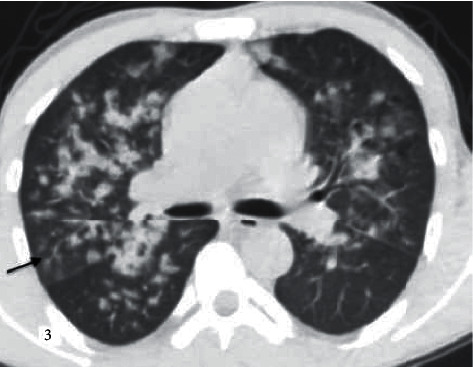
A 31-year-old male patient with influenza B pneumonia was also diagnosed with known end-stage renal disease. The score was evaluated as 2 according to CORADS classification and in the atypical group according to the RSNA guidelines. Soft tissue density centrilobular nodules (black arrow) forming tree in bud pattern and peribronchovascular consolidation.

**Figure 4:**
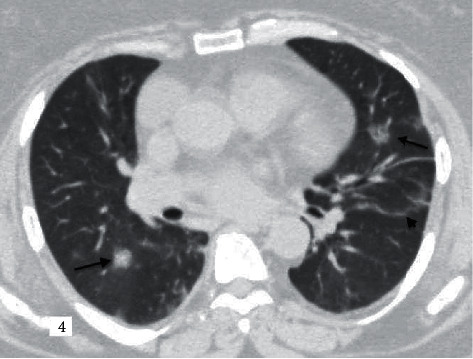
A 60-year-old female patient with influenza A (H1N1) pneumonia, known diabetes, and chronic kidney disease. According to the RSNA guidelines in the typical group, CORADS score is given as 5. Bilateral rounded consolidation areas (black arrows) and parenchymal band (black arrowhead) are observed.

**Figure 5:**
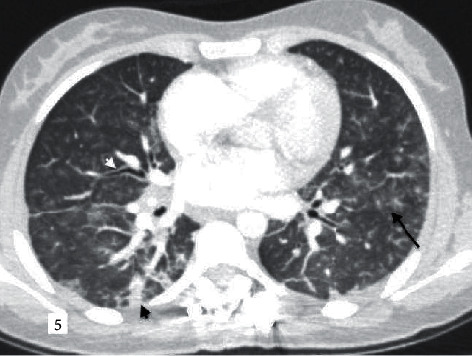
An 18-year-old female patient with parainfluenza (HPIV 3) pneumonia also with bone marrow transplantation due to acute lymphoblastic leukemia. According to the RSNA guidelines in “indetermine,” CORADS score given as 3. Diffuse centrilobular ground glass density nodules (black arrow), focal peripheral consolidation areas (black arrowhead), and increased peribronchial wall thickness (white arrowhead) are observed.

**Figure 6:**
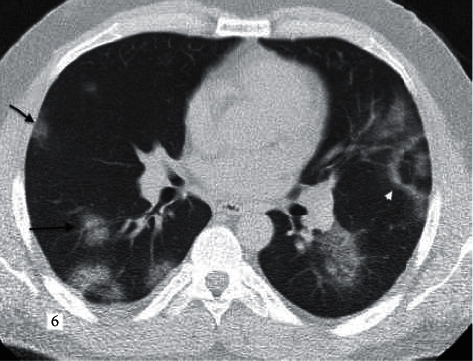
A 32-year-old male patient with COVID-19 pneumonia with a known diagnosis of asthma. Typical presentation according to RSNA guidelines, CORADS score given as 5. Bilateral lung parenchyma rounded, multifocal GGO lesions (black arrows), reversed halo sign (white arrow) center is relatively normal, with GGO in the periphery.

While COVID-19 pneumonia often involves peripheral, central, or random multilobar distribution with peribronchovascular, pure consolidation is observed in influenza and pneumonia (Figures 2–6). In addition, it is reported that the presence of round opacities, interlobular septal thickenings, crazy paving, sharper lesion margin, and the absence of nodules or tree in bud appearance are helpful features for COVID-19 pneumonia to distinguish it from influenza [13, 15, 16].

**Figure 7:**
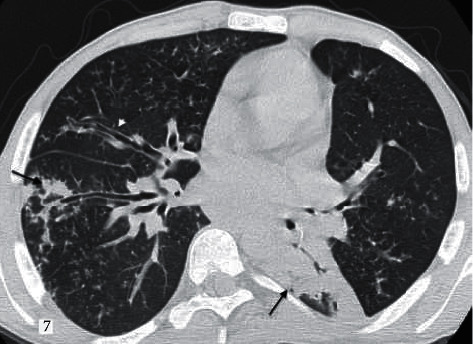
A 24-year-old male patient diagnosed with known primary immunodeficiency with adenovirus pneumonia. According to the RSNA guidelines “undetermined,” CORADS score given as 4. Irregular peripheral consolidation (black arrows) and increased peribronchial thickness (white arrowhead) are observed.

Current literature suggests that the pulmonary target sign, which is defined as a variant of the reversed halo sign by making a difference with the hyperdense dot sign in the center, is diagnostic in COVID-19 viral pneumonia [17, 18]. In our study, we did not evaluate the presence of a central hyperdense dot as a separate parameter. However, the presence of the reversed halo sign is valuable in differentiating other viral pneumonia from COVID-19 (Figure 6). Furthermore, studies have reported that CT findings of adenovirus pneumonia and COVID-19 pneumonia (segmental and subpleural consolidations, air bronchogram, interlobular septal thickening, accompanying mildly limited GGO, and pleural effusion) overlap [19] (Figure 7).

Classification recommendations such as the RSNA consensus guideline [6] and CORADS [20, 21] have been brought to the agenda in the pandemic process with the aim of investigating COVID-19 pneumonia imaging findings by standardized means to ensure a universal reporting language can be used in communication with other branches for patient management. Studies evaluating the diagnostic performance of CORADS report a consistent evaluation system with high positive predictions [7, 22–25]. According to the RSNA consensus guidelines, the scores of the atypical group and CORADS 2, and the indetermined group and CORADS 3 correspond to each other and were found to be significant in favour of non-COVID-19 viral pneumonia. The RSNA typical group and the CORADS 5 score also correspond to each other and were found to be similarly significant in favour of COVID-19 pneumonia. It is likely that the lack of diagnostic difference between the CORADS 4 score groups may be due to the fact that frequent findings in other viral pneumonia such as small but peripherally localized unilateral GGO and multifocal consolidation without other typical findings are included in this category. Although it has been reported that dividing the RSNA indeterminate category into 3 and 4 in the CORADS system limits intraobserver variability [7], these assessment systems were developed during the pandemic process; so, when the prevalence of COVID-19 decreases after the pandemic is over, this issue that needs evaluation for how it can be applied to incidental thoracic CT findings independent of the clinic and in the future, these studies may contribute to improving the diagnostic efficiency of CORADS.

A limitation of this study was that retrospective design caused a discrepancy between the numbers of patients with COVID-19 and non-COVID-19 viral pneumonia. Unlike COVID-19 pneumonia, chest CT was rarely utilized in the diagnostic workup of patients with viral pneumonia in the past. Our results may have been affected by biases secondary to confounding factors. We could not do propensity matching in this study due to the small number of patients in the COVID-19 group which would have further reduced with propensity matching. Secondly, the time interval between the CT scans and PCR tests in patients in the non-COVID-19 group was relatively longer (5.67 ± 7.95 days) compared to the COVID-19 group which may have affected the CT scan findings. Thirdly, the presence of coinfection in patients diagnosed with COVID-19 pneumonia is unknown since most of these patients did not have additional other (bacterial or fungal) microbial culture examinations during the pandemic. Nonetheless, we expect hospital-acquired coinfection to be lower since we have evaluated the first thorax CT examinations of these patients diagnosed with COVID-19 pneumonia.

In conclusion, for the diagnosis of viral pneumonia, radiological imaging evaluated together with laboratory examinations and particularly clinical and gold-standard RT-PCR tests has an important role in diagnosis and patient management. RSNA classification and CORADS scoring system can be used successfully to distinguish COVID-19 pneumonia from non-COVID-19 pneumonia. The presence of reversed halo sign and absence of pleural effusion was found to be efficient in the diagnosis of COVID-19 pneumonia.

## Figures and Tables

**Table 1:** Distribution of patients according to age, gender, and concomitant diseases.

	Non-COVID-19	COVID-19	All patients
Averages of ages and standard deviation	49.29 ± 19.43	53.01 ± 15.91	51.99 ± 16.99

Min-max (median)	18–84 (53.5)	18–91 (53)	18–91 (53)

Gender			
Female (%)	41.5 (*n* = 39)	40.2 (*n* = 100)	40.5 (*n* = 139)
Male (%)	58.5 (*n* = 55)	59.8 (*n* = 149)	59.5 (*n* = 204)

Concomitant chronic illness			
Absent (%)	—	33 (*n* = 113)	
Cardiovascular disease (%)	3.7 (*n* = 4)	4.1 (*n* = 14)	
Hypertension (%)	1.9 (*n* = 2)	22.5 (*n* = 77)	
Diabetes mellitus (%)	5.6 (*n* = 6)	14.6 (*n* = 50)	
Chronic lung disease (%)	2.8 (*n* = 3)	1.8 (*n* = 6)	
Chronic liver disease (%)	1.9 (*n* = 2)	0 (*n* = 0)	
Chronic kidney disease (%)	19.4 (*n* = 21)	2.3 (*n* = 8)	
Malignancy (extrapulmonary) (%)	21.3 (*n* = 23)	3.2 (*n* = 11)	
Conditions related to immunodeficiency (%)	28.7 (*n* = 31)	3.5 (*n* = 12)	
Others (%)	14.8 (*n* = 16)	14.9 (*n* = 51)	

**Table 2:** Chi-square test analysis findings in relation between non-COVID-19 and COVID-19 groups in terms of RSNA consensus guide classification, CORADS scoring, and imaging findings of thorax computed tomography.

		Group		*p*
RSNA consensus		Non-COVID-19	COVID-19	0.001^*∗∗*^
	Typical Indetermine Atypical	38 (40.4%)	214 (85.9%)	
		32 (34%)	29 (11.7%)	
		24 (25.5%)	6 (2.4%)	
CORADS	CORADS 2/low	27 (28.7%)	7 (2.8%)	
	CORADS 3/Indetermine	30 (31.9%)	17 (6.8%)	
	CORADS 4/high	12 (12.8%)	31 (12.4%)	
	CORADS 5/very high	25 (26.6%)	194 (77.9%)	
Percentage of involvement	%0–%25	32 (34%)	107 (43%)	
	%25–%50	29 (30.9%)	97 (39%)	
	%50–%75	17 (18.1%)	34 (13.7%)	
	%75<	16 (17%)	11 (4.4%)	
Number of lesions	Single	1 (1.1%)	17 (6.8%)	**0.022** ^*∗*^
	Multiple	93 (98.9%)	232 (93.2%)	
Distribution preference	Peripheral	11 (11.7%)	101 (40.7%)	**0.001** ^*∗∗*^
	Central	2 (2.1%)	2 (0.8%)	
	Peripheral + central	81 (86.2%)	145 (58.5%)	
Dominant pattern	GGO	53 (56.4%)	196 (78.7%)	**0.001** ^*∗∗*^
	Consolidation	13 (13.8%)	46 (18.5%)	
	Linear, reticular opacity	2 (2.1%)	3 (1.2%)	
	Nodule	26 (27.7%)	4 (1.6%)	

		*Group*		*p*

Distribution pattern of GGO		Non-COVID-19	COVID-19	**0.001** ^*∗∗*^
	Absent	10 (10.6%)	3 (1.2%)	
	Peripheral-bilateral	19 (20.2%)	140 (56.2%)	
	Round-multifocal	20 (21.3%)	68 (27.3%)	
	Halo sign	1 (1.1%)	0 (0%)	
	Diffuse	26 (27.7%)	4 (1.6%)	
	Perihilar-not round	5 (5.3%)	3 (1.2%)	
	Single-sided-not round	13 (13.8%)	31 (12.4%)	
Nodule		56 (60.6%)	12 (4.8%)	**0.001** ^*∗∗*^
Tree in bud pattern		49 (52.1%)	8 (3.2%)	**0.001** ^*∗∗*^
Interstitial changes	Absent	24 (25.5%)	60 (24.1%)	**0.001** ^*∗∗*^
	Septal thickening	33 (35.1%)	17 (6.8%)	
	Fine reticular opacity	7 (7.4%)	72 (28.9%)	
	Septal thickening + fine reticular opacity	30 (31.9%)	100 (40.2%)	
“Crazy paving” pattern		13 (13.8%)	76 (30.5%)	**0.001** ^*∗∗*^
Reversed halo (Atoll)		6 (6.4%)	109 (43.8%)	**0.001** ^*∗∗*^
Microvascular enlargement		60 (63.8%)	207 (83.1%)	**0.001** ^*∗∗*^
Linear opacities		71 (75.5%)	157 (63.1%)	**0.029** ^*∗*^
Traction bronchiectasis		45 (47.9%)	155 (62.2%)	**0.016** ^*∗*^

		Non-COVID-19	COVID-19	*p*
Peribronchial wall thickening		55 (58.5%)	82 (32.9%)	**0.001** ^*∗∗*^
Air trapping		31 (33%)	29 (11.6%)	**0.001** ^*∗∗*^
Pleural retraction		54 (57.4%)	99 (39.8%)	**0.001** ^*∗∗*^
Pleural effusion		31 (33%)	8 (3.2%)	**0.001** ^*∗∗*^
Pericardial effusion		28 (29.8%)	9 (3.6%)	**0.001** ^*∗∗*^
Cavitation		3 (3.2%)	0 (0%)	**0.001** ^*∗∗*^
Mediastinal-hilar lymph node	Nonspecific	65 (69.1%)	229 (92%)	**0.001** ^*∗∗*^
	Pathological	28 (29.8%)	20 (8%)	
	Another reason	1 (1%)	0 (0%)	
Dominant lesion size	0–3 cm	43 (45.7%)	85 (34.1%)	0.122
	3–5 cm	10 (10.6%)	46 (18.5%)	
	5–7 cm	8 (8.5%)	30 (12%)	
	>7 cm	33 (35.1%)	88 (35.3%)	
Consolidation		52 (55.3%)	137 (55%)	0.960
Vacuolar sign		11 (11.7%)	22 (8.8%)	0.270
Halo sign		19 (20.2%)	60 (24.2%)	0.436
Subpleural curvilinear opacity		23 (24.5%)	75 (30.1%)	0.301
Air bronchogram		23 (24.5%)	49 (19.8%)	0.340
Pleural thickening		19 (20.2%)	54 (21.7%)	0.766

Chi-square test, ^*∗∗*^*p* < 0.01.

**Table 3:** Multiple regression analysis findings in relation to independent variables to COVID-19.

Model	Variables	*B*	S. error	*β*	*p*
1	Constant	1.078	115344.8	2.939	0.999
	Percentage of involvement, 0%–25%	−0.512	1.538	0.599	0.739
	Percentage of involvement, 25%–50%	0.07	1.207	1.073	0.954
	Percentage of involvement, 50%–75%	−0.962	1.12	0.382	0.39
	Percentage of Involvement, <75%	−0.714	1.028	0.49	0.487
	Number of lesions, single	−18.856	8494.375	0	0.998
	Transverse distribution, peripheral	13.089	40192.85	483558.2	0.999
	Transverse distribution, central	7.533	40192.85	1869.159	0.999
	Transverse distribution, peripheral + central	13.342	40192.85	622821.2	0.999
	Dominant pattern, GGO	−22.783	22512.07	0	0.999
	Dominant pattern, consolidation	−26.04	22512.07	0	0.999
	Dominant pattern, linear, reticular opacity	−23.08	22512.07	0	0.999
	Dominant pattern, nodule	−23.966	22512.07	0	0.999
	GGO, peripheral-bilateral	1.083	1.363	2.955	0.427
	GGO, round-multifocal	−1.24	1.023	0.289	0.225
	GGO, halo sign	0.377	1.066	1.457	0.724
	GGO, diffuse	22.949	9516.478	9.26*E* + 09	0.998
	GGO, perihilar-not round	1.507	1.34	4.514	0.261
	GGO, single-sided-not round	1.533	6.119	4.633	0.802
	Nodule	2.308	1.307	10.052	0.078
	Tree in bud pattern	1.316	1.354	3.727	0.331
	Interstitial changes, absent	0.231	0.958	1.26	0.809
	Interstitial changes, septal thickening	1.021	0.826	2.777	0.216
	Interstitial changes, fine reticular opacity	−0.463	0.87	0.63	0.595
	Crazy paving pattern	−0.454	0.833	0.635	0.586
	**Reversed halo**	−**2.334**	**0.952**	**0.097**	0.014^*∗*^
	Microvascular enlargement	−0.203	0.614	0.816	0.741
	Linear opacities	−0.73	0.733	0.482	0.319
	Traction bronchiectasis	−0.23	0.603	0.794	0.703
	Peribronchial wall thickening	0.561	0.512	1.753	0.273
	Air trapping	1.222	0.621	3.394	0.055
	Pleural retraction	1.063	0.614	2.895	0.083
	**Pleural effusion**	**2.364**	**0.743**	**10.631**	0.001^*∗∗*^
	Pericardial effusion	0.774	0.722	2.168	0.284
	Cavitation	44.133	19385.62	1.47*E* + 19	0.998
	Mediastinal-hilar lymph node, nonspecific	−3.993	41304.74	0.018	0.999
	Mediastinal-hilar lymph node, pathological	−3.184	41304.74	0.041	0.999

*R* ^2^ = 0.793; *X*^2^_(1.51)_ = 130,451; *p*=0.001^*∗∗*^; ^*∗*^*p* < 0.05; and ^*∗∗*^*p* < 0.01.

## Data Availability

The data used to support the findings of this study may be released upon application to the [Istanbul Faculty of Medicine, Clinical Research Ethics Committee], who can be contacted at [itfetikkurul@istanbul.edu.tr].
